# Cost-Effective Full-Color 3D Dental Imaging Based on Close-Range Photogrammetry

**DOI:** 10.3390/bioengineering10111268

**Published:** 2023-10-31

**Authors:** Bin Yang, Jennifer Schinke, Amir Rastegar, Melikhan Tanyeri, John A. Viator

**Affiliations:** Department of Biomedical Engineering, Duquesne University, Pittsburgh, PA 15282, USA; schinkej@duq.edu (J.S.); rastegara@duq.edu (A.R.); tanyerim@duq.edu (M.T.); viatorj@duq.edu (J.A.V.)

**Keywords:** photogrammetry, dental imaging, 3D dental reconstruction, intraoral scanner

## Abstract

Dental imaging plays a crucial role in clinical dental practice. Conventional 2D dental imaging serves general-purpose tasks, such as patient documentation, while high-precision 3D dental scanning is tailored for specialized procedures, such as orthodontics and implant surgeries. In this study, we aimed to develop a cost-effective 3D imaging technique that could bridge the gap between conventional dental photography and high-precision 3D dental scanning, with the goal of improving patient dental care. We developed a 3D imaging technique based on close-range photogrammetry and termed it close-range photogrammetry-based dental imaging (CPDI). We evaluated this technique on both in vitro dental models and in vivo teeth. For dental models, we conducted a parametric study to examine the effects of the depth of field and specular reflection on reconstruction quality. We showed that the optimal results were achieved with an f/5.6 lens and without a circular polarizer for reflection suppression. This configuration generated 3D scans with 57.7 ± 3.2% and 82.4 ± 2.7% of reconstructed points falling within ±0.1 mm and ±0.2 mm error margins, respectively. With such accuracy, these 3D dental models can faithfully represent dental morphology and features. During in vivo imaging, we were able to reconstruct high-quality 3D models of the anterior arch, further demonstrating its clinical relevance. The reconstructed models carry both 3D shapes and detail full-color surface textures, which positions CPDI as a versatile imaging tool in different areas of clinical dental care.

## 1. Introduction

Dental photography serves as an effective way for documentation, patient communication and education, and treatment planning in modern dental practice [1,2,3,4]. While conventional dental photography captures images in high spatial resolution and in color, it is limited to providing 2D information. In certain cases, without a comprehensive 3D representation, specific dental conditions may necessitate the capture of multiple photos from different angles, thus complicating diagnosis and treatment. In addition to dental photography, 3D dental imaging is also available [5,6]. Applications with 3D dental imaging tend to be more specialized areas, such as orthodontics and dental implants. Within orthodontics, 3D scanning has become a standard procedure for assessment, treatment planning, and the fabrication of dental aligners [7,8]. In dental implant surgeries, the use of 3D imaging improves surgical outcomes by reducing potential complications and enabling more precise implant placement [9,10,11].

Several imaging techniques have been developed for dental imaging, including structured light and stereo vision [6,12]. Structured light uses active illumination to project patterns, such as coded stripes and speckles, onto the surface [13,14]. Due to the surface elevation (3D shape), these patterns are deformed, and this deformation is analyzed to reconstruct the 3D shape of the scene. On the other hand, stereo vision utilizes two cameras to image the scene. Due to parallax, identical features are captured at different locations on the two sensors [6,15]. This positional difference on the sensor is known as disparity, which is utilized to determine the depth [16,17]. While these imaging techniques provide excellent results for orthodontics and implant surgeries, the cost and complexity of the technology probably do not justify adopting such methods for other general imaging needs in dental care.

In recent years, close-range photogrammetry has emerged as an appealing alternative to the existing 3D imaging technologies [18,19,20]. Close-range photogrammetry does not require active illumination and only needs a single camera. It can generate full-color 3D models from a set of images or a video. Photogrammetry estimates the 3D positions of points by triangulating common features found in multiple images [21]. Due to its straightforward system setup and ease of use, close-range photogrammetry-based dental imaging (CPDI) has shown great potential in dental practice and has undergone extensive exploration [22,23,24]. Notably, the majority of CPDI has been performed on plaster cast dental models typically with a professional digital camera. Data on in vivo CDPI are limited, possibly due to the lack of appropriate intraoral imaging devices. In this study, we focused on (1) demonstrating and evaluating the capability of CDPI to generate 3D dental models in an in vivo setting by (2) developing and testing a compact and cost-effective intraoral camera. An affordable camera would lower the barrier for adopting this technology, potentially improving the quality of patient care.

## 2. Materials and Methods

### 2.1. Imaging Device Development

**Figure 1:**
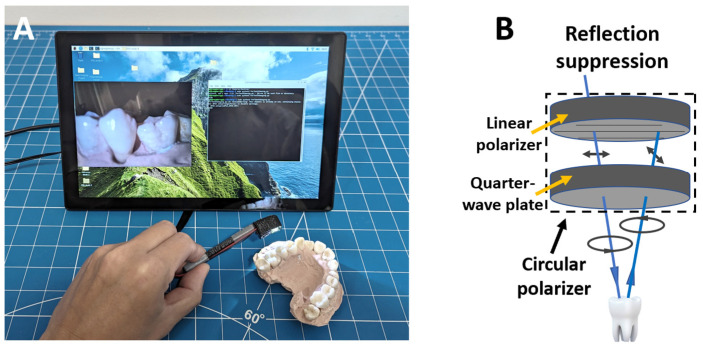
(**A**) Raspberry-Pi-based dental imaging system. (**B**) Working principle of reflection suppression using a circular polarizer.

We developed two low-cost imaging setups based on a Raspberry Pi 4 (RPi) single-board computer (1) to conduct parametric studies of the effects of depth-of-field and surface reflection on reconstruction and (2) to evaluate the performance of in vivo teeth imaging. A typical configuration of the imaging system is shown in Figure 1A. For parametric studies, we adopted an RPi-compatible camera module featuring an M12 mount (UR-261, Arducam, Nanjing, China). This camera is later referred to as the M12 camera. Although this camera type is typically large and not ideal for in vivo imaging, its standard M12 mount allows for the use of a variety of M12 camera lenses. Illumination was provided by two 1 W white LEDs positioned on both sides of the camera. To reduce stray light, two flexible light-blocking sheets were placed between the LED and the camera. A more compact setup was developed for in vivo imaging, which comprised a compact Rpi imaging module (Module V2, Raspberry Pi, Cambridge, UK) with a fixed lens and a single 1 W white LED for illumination. The imaging setup was assembled into a toothbrush-like structure with a compact imaging head (2 × 1 × 1 cm, L × W × D). This camera is later referred to as the intraoral camera. Both imaging setups utilized the same 8 MP imaging sensor (IMX 219, SONY, Tokyo, Japan). Circular polarizers could be added to both imaging modules to reduce specular reflection, given that both illumination and imaging share a common optical path [25]. Figure 1B illustrates the principle of reflection suppression with a circular polarizer. A circular polarizer consists of a linear polarizer and a quarter-wave plate. The quarter-wave plate introduces a 90 degree phase shift to the polarized light [26]. In the incident direction, the 90 degree phase shift turns the linear polarized light into circular polarized light. Upon reflection from the teeth surface, the handedness of the circular polarized light is reversed. The 90 degree phase shift from the quarter-wave plate converts the reflected light into linear polarized light with the polarization direction 90 degrees apart from the original one, which is blocked by the linear polarizer. A custom Python script was developed to adjust imaging parameters and capture images. To ensure high-quality and consistent images, we used a fixed white balance setting and an ISO value of 200. Throughout imaging, an auto-exposure setting was used with a fixed exposure compensation value to reduce the risk of overexposure.

### 2.2. Parametric Studies of the Effects of Depth-of-Field and Surface Reflection on Reconstruction Quality

Intraoral imaging poses several challenges, such as a short working distance and surface reflection, that can potentially impact the quality of the 3D reconstruction. The parametric study was designed to understand these challenges and optimize imaging parameters. The working distance of intraoral imaging needs to be short to accommodate the limited space in the oral cavity. A shorter working distance will result in a shallower depth-of-field (DoF), which means a smaller portion of the object will remain in focus. A lens with a smaller aperture can be used to alleviate this issue at the expense of lower light collection efficiency, potentially affecting the image quality. To understand how DoF affects the reconstruction quality, we imaged a dental model using the M12 camera with two lenses sharing the same focal length but differing in aperture sizes. The F/2.8 lens (89342, Edmund Optics, Barrington, NJ, USA) features a larger aperture, producing a shallower DoF, while the F/5.6 lens (89343, Edmund Optics, Barrington, NJ, USA) has a smaller aperture, resulting in a more moderate DoF. The enamel reflects light strongly under illumination, often leading to image saturation and subsequent loss of information. To study whether the suppression of reflected light can improve the reconstruction quality, we imaged the same dental model both with and without a circular polarizer (88-085, Edmund Optics, Barrington, NJ, USA). A circular polarizer effectively reduces reflection when the illumination and imaging share a common optical path. For each testing condition, the dental model was imaged three times.

### 2.3. In Vivo Teeth Imaging

The anterior sections of both the upper and lower arches of a volunteer were imaged using an intraoral camera. Prior to imaging, the volunteer brushed the teeth, and excess fluids on the teeth were wiped with gauze. The intraoral camera was positioned approximately 2 cm in front of the teeth and followed the curvature of the arch during image acquisition. Each portion of the arch was imaged from multiple angles with a good focus, which was visually confirmed before taking the picture. It is worth noting that the imaging was performed by the same volunteer without the need for additional assistance, further demonstrating the user-friendly nature of this imaging technique. Due to the specular reflections from the moist teeth surface, a circular polarizer (88-085, Edmund Optics, Barrington, NJ, USA) was used throughout the in vivo teeth imaging to mitigate these reflections. Images were subsequently reconstructed for visualization.

### 2.4. Photogrammetry-Based 3D Reconstruction

Photogrammetry, as a versatile 3D reconstruction technique, has been widely used in remote sensing and aerial applications [27,28,29], archaeology [30], and augmented/virtual reality applications [31]. Detailed descriptions of the working principles of photogrammetry are beyond the scope of this study and have been well documented elsewhere [20,30]. Briefly, photogrammetry involves capturing a set of images of the subject from various directions. Common features of the subject within a subset of the images are detected and extracted using certain techniques, such as the scale-invariant feature transformation (SIFT) method [32]. These detected features are then used to estimate the 3D coordinates of the features based on triangulation [21]. The point cloud of the subject is generated after the 3D coordinates of all features have been estimated. This point cloud is further processed, meshed, and textured to create a faithful 3D representation of the subject.

**Figure 2:**
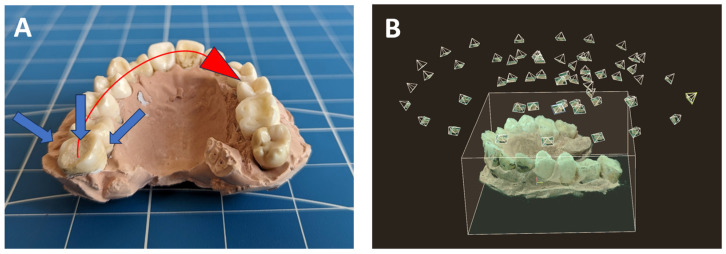
(**A**) Image acquisition paths following the curvature of the arch (red arrow) at different angles (blue arrows) for complete coverage. (**B**) Visualization of camera positions and angles.

In practice, photogrammetry requires that images have sufficient overlap to facilitate feature detection, registration, and reconstruction [20]. In both parametric studies and in vivo imaging, we maintained approximately 50% image overlap for all acquired images. As illustrated in Figure 2A, we followed specific imaging paths to ensure comprehensive coverage from various angles. The camera was positioned sequentially at approximately 45, 90, and 135 degrees towards the dental model (indicated by blue arrows in Figure 2A). At each angle, images were taken while following the curvature of the arch, as depicted by the red arrow. Figure 2B visualizes the camera positions and angles during a CPDI session. For the parametric study, 30–40 images were acquired to reconstruct the full arch, while in in vivo imaging, 20–30 images were acquired.

### 2.5. Reconstruction and Trueness Analysis

Following image acquisition, we reconstructed 3D models using PhotoCatch (EOS Innovations LLC) running on a M1 Mac Mini. The reconstructed 3D model was exported in .obj format, which contains both the 3D structure mesh and color texture of the dental model. The 3D model was imported into CloudCompare (V 2.12.4), an open-source point processing software, for visualization and trueness analysis.

To facilitate the trueness analysis, the teeth portion of the reconstructed dental model was manually segmented out in CloudCompare. Due to the lack of a physical dimension of the 3D model generated with the photogrammetry technique, the initial alignment between the 3D teeth model and the ground truth model was performed manually. Specifically, the 3D teeth model was manually scaled to the approximate size of the ground truth mode. Following scaling, the orientation of the 3D teeth model was adjusted to match the orientation of the ground truth model. Once a good manual alignment between these two models was achieved, we utilized the registration function provided in CloudCompare for a more precise registration. During the registration process, we designated the ground truth as the target and allowed the 3D teeth model to be further scaled to minimize the overall error between the two models. Unlike CPDI, the ground truth model was generated on an absolute scale using a commercial intraoral scanner. Local deviations, measured in millimeters, between the ground truth model and the reconstructed model were quantified to indicate the reconstruction errors. As the ground truth for the in vivo dental model was not available, trueness analysis was not conducted for in vivo imaging.

## 3. Results

### 3.1. Imaging Device Development

**Figure 3:**
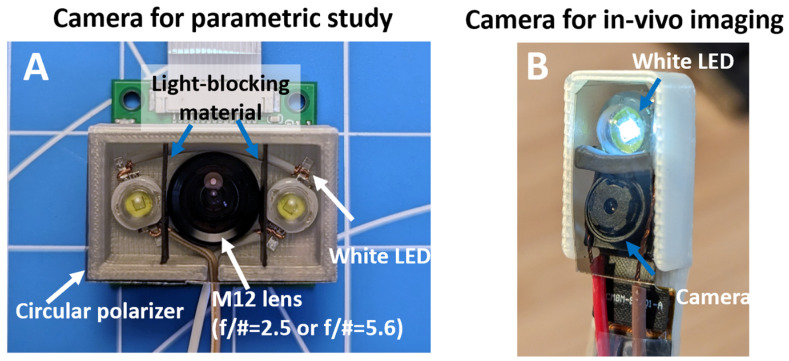
(**A**) Camera setup for parametric study with interchangeable M12 lens. (**B**) Toothbrush-like compact camera for in vivo imaging. Both cameras can be equipped with a circular polarizer to suppress surface reflection.

Both the M12 camera for parametric studies (Figure 3A) and the in vivo intraoral camera (Figure 3B) share a similar configuration; however, the intraoral camera is noticeably smaller. The lens on both devices was adjusted to achieve a short working distance of approximately 1.5 to 2 cm. The horizontal field-of-view (FoV) is about 70 degrees for both cameras.

### 3.2. Parametric Studies of the Effects of Depth-of-Field and Surface Reflection on Reconstruction Quality

**Figure 4:**
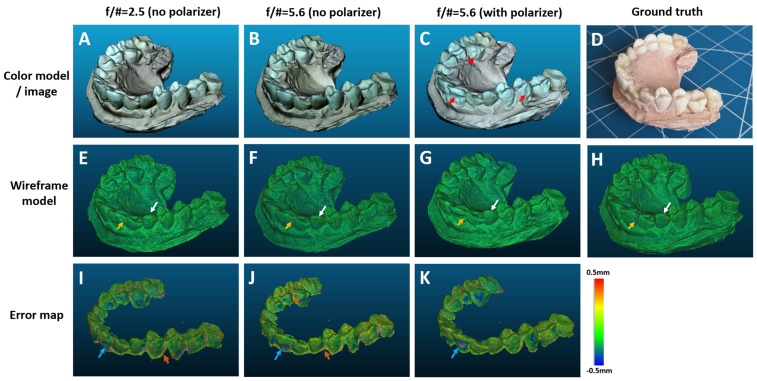
Parametric study of CPDI under three imaging settings: f/2.5 lens without circular polarizer (**A**,**E**,**I**), f/5.6 lens without circular polarizer (**B**,**F**,**J**), and f/5.6 lens with circular polarizer (**C**,**G**,**K**). Results were visualized in full color (**A**–**C**) and in wireframe (**E**,**F**). The reconstruction error was quantified and visualized (**I**–**K**) against the ground truth model (**H**). A color picture of the dental model is shown in (**D**).

**Figure 5:**
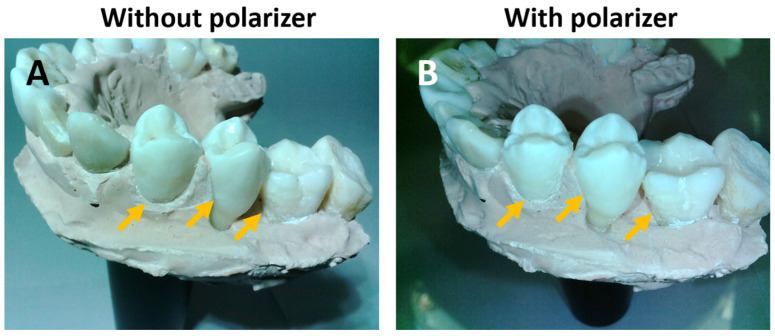
A circular polarizer reduces the surface contrast of a dental model (**B**) compared to that without a polarizer (**A**) as highlighted with yellow arrows.

The reconstructed 3D dental models under three camera configurations (Figure 4A–C) exhibited an excellent and detailed visual representation of the physical dental model (Figure 4D). The colors of the teeth and the plaster base were faithfully reproduced along with their surface textures. The morphology of the incisor, pre-molar, and molar teeth are distinctly shown in the reconstructed model. The models generated with f/2.5 (Figure 4A) and f/5.6 (Figure 4B) lenses, both without a polarizer, are visually comparable. However, the model obtained with a polarizer in combination with an f/5.6 lens (Figure 4C) showed noticeable reconstruction errors, as indicated by the red arrows, unlike the models obtained without a polarizer (Figure 4A,B). This result was surprising, given that the polarizer typically improves the image quality by reducing surface glare. Comparing images acquired with and without a polarizer, we noticed that the image acquired with a polarizer showed reduced contrast in certain regions, as indicated by the orange arrows (Figure 5). During the interaction between the polarized light and the teeth, reflection occurs both at the tooth surface and within the deeper layers (diffuse reflection). The surface reflection carries crucial surface contrast information necessary for the reconstruction process. However, the circular polarized light reflected from the surface exhibited a reversed handedness, which was suppressed by the circular polarizer in its return path. As a result, the surface contrast information was reduced, which may have adversely affected the reconstruction accuracy.

Comparing the reconstructed 3D dental models (Figure 4E–G) to the ground true model (Figure 4H) in wireframe mode effectively eliminates potential interference from color texture. It is evident that the ground truth model was reproduced with a higher accuracy, exhibiting a higher level of detail with sharper edges and smoother surfaces. For instance, while the top surfaces of one incisor (indicated with a white arrow) are small, they are clearly visible in the ground truth model. The three reconstructed models failed to capture the same surface features. Similarly, the individual teeth are more distinctly separated with clearly defined gaps in the ground truth model, as opposed to the reconstructed models (indicated by yellow arrows).

The error maps generated from the quantitative trueness analysis aligned well with the results of the visual inspection (Figure 4I–K). Figure 4I–K reveal that areas with high reconstruction errors typically appear in the recessed regions (gaps) (indicated by orange arrows) or on the smooth surfaces (indicated by cyan arrows).

**Figure 6:**
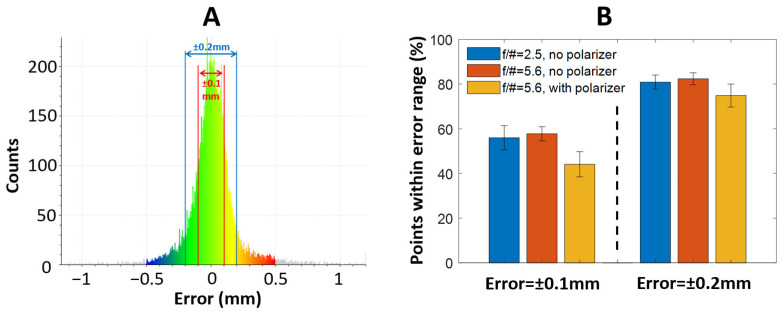
(**A**) Histogram of the reconstruction error with f/5.6 lens and without polarizer. (**B**) Percentage of reconstructed points within the error range of ±0.1 mm and ±0.2 mm for three imaging settings. The error bar represents the standard deviation over 3 trials.

Trueness was quantified within two error ranges: ±0.1 mm and ±0.2 mm. An accuracy of 0.1 mm is generally accepted to be clinically adequate and has been used for evaluating the performance of conventional intraoral scanners [33]. A typical histogram of the reconstruction error is shown in Figure 6A. It is evident that the majority of the reconstructed points fall within the ±0.2 mm error range. Notably, some reconstructed points exhibit a larger error (>0.5 mm, shown in gray color). Without a polarizer, the f/2.5 and f/5.6 lenses achieved averages of 55.9 ± 5.4% and 57.7 ± 3.2% of the reconstructed points within the ±0.1 mm error range. The extended DoF offered by the f/5.6 lens resulted in a minor improvement in reconstruction quality. With the f/5.6 lens, the reconstruction accuracy was notably higher without a polarizer than that achieved with a polarizer, which was 44.1 ± 5.6%. When relaxing the error to ±0.2 mm, the f/2.5 and f/5.6 lenses without a polarizer achieved an averaged inlier ratio of 80.9 ± 3.1% and 82.4 ± 2.7%, which was approximately a 25% improvement over the ±0.1 mm error range. A slightly greater improvement in the inlier ratio (74.9 ± 5.2%) was realized using an f/5.6 lens and a polarizer. Overall, the reconstruction with an f/5.6 lens without a polarizer yielded the most accurate and consistent results.

### 3.3. In Vivo Teeth Imaging

**Figure 7:**
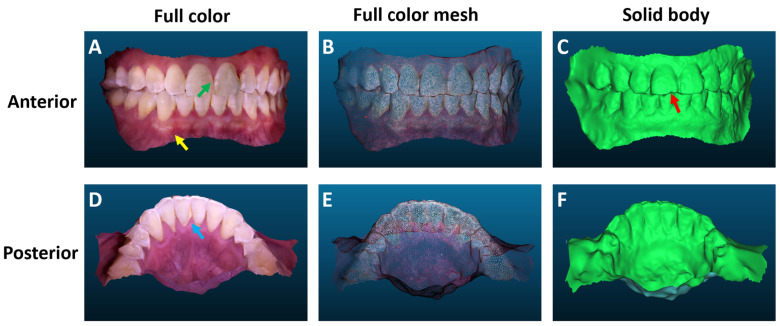
Full-color 3D models of (**A**) upper and lower labial arches (anterior view) and (**D**) lower labial arch (posterior view). (**B**,**E**) show the corresponding full-color meshes, and (**C**,**F**) show the solid body of the reconstructed arches. The green, yellow, and cyan arrows show tooth discoloration, gum, and dental plaque, respectively. The red arrow shows a small chip in the incisor tooth.

The in vivo teeth imaging demonstrated that both the color and 3D shape of teeth can be reproduced with high fidelity with CPDI. The anterior (Figure 7A) and posterior (Figure 7B) views of the full-color 3D models effectively captured the volunteer’s intraoral condition. It is worth noting that the gums were also reconstructed as part of the 3D model. Owing to the nature of the image-based reconstruction, the 3D model accurately preserved colors and intricate features, which could enable a comprehensive evaluation of dental health and the examination of gum-related conditions. For instance, the discoloration of the incisor teeth (indicated by the green arrow in Figure 7A), small blood vessels in the gums (indicated by the yellow arrow in Figure 7A), and dental calculus (indicated by the cyan arrow in Figure 7D) were all visible in high resolution. The full-color meshes (Figure 7B,E) revealed the 3D models were densely reconstructed. In the solid body view, individual teeth were reconstructed with precise morphological features and clear separation from neighboring teeth. For instance, a small chip of an incisor tooth is clearly visible (indicated by the red arrow in Figure 7C). Some imperfections, such as non-smooth surfaces, were noticeable in the solid model. However, these imperfections did not significantly impact the visualization of the teeth and gums, particularly with the full-color models. Despite the lack of a ground truth model of the volunteer for quantitative analysis, it is evident, based on the visual inspection, that CPDI is effective in in vivo 3D dental imaging.

## 4. Discussion

CPDI aims to fill the gap between conventional 2D dental photography and high-precision 3D dental scanning and to improve the quality of patient care. Similar to 2D dental photography, CPDI provides rich and accurate color information in high spatial resolution, facilitating detailed close-up examinations. Furthermore, this color information is integrated into the 3D dental model, allowing it to be visualized and evaluated from arbitrary angles with the awareness of its 3D geometry. It is worth noting that while CPDI-derived models may not achieve the precision of those obtained with commercial 3D dental scanners, they faithfully reconstruct the most prominent morphological and structural dental features of the dental arch and individual teeth. This compromise is justified as CPDI is not intended for orthodontics and dental surgeries where a highly precise dental scan is critical to ensure optimal treatment outcomes. The low-cost nature of CPDI could lower the barrier for adopting this technology in clinical dental practice, potentially improving the quality of patient care.

The parametric study suggested that while the surface reflection is undesirable in an image, it appears not to significantly affect the reconstruction results. This is potentially because the same region exhibiting high reflection in one image may not do so in other images due to the change in imaging angles. With the information from multiple images, those regions can still be reconstructed. Suppressing reflection seems to deteriorate the reconstruction quality, possibly due to the loss of surface contrast, as discussed in Section 3.2. In the in vivo imaging, we had to use a polarizer to mitigate excessive reflection. The reconstruction quality, however, was better than the result presented in the parametric study with a polarizer. While the exact cause warrants further investigation, we speculate that sufficient surface contrast of the teeth was retained for this specific volunteer, even with a polarizer. This also suggests that whether or not to use a polarizer for surface reflection suppression needs to be tested and evaluated for each imaging task. A lens with a smaller aperture (offering a larger DoF) indeed improved the overall reconstruction quality, but only by a small margin, suggesting that such a lens is still preferable under adequate illumination. If illumination intensity and potential motion blur become a point of concern, opting for a lens with a larger aperture would still yield satisfactory results.

Through in vivo 3D imaging, we demonstrated accurate reconstructions of both the incisor and canine teeth. However, it proved challenging to reconstruct the pre-molar and molar teeth due to certain limitations within the current system. These limitations, in turn, provide valuable insights for guiding future improvements. Despite the overall compact size of the intraoral camera, the working distance is still too large to comfortably image the teeth positioned deep within the oral cavity. A potential improvement could be achieved by introducing a variable extension tube to the imaging system. This adaptation will allow easy adjustment of the working distance and FoV to facilitate imaging different portions of the arch, such as a larger working distance (larger FoV) for imaging the anterior arch and a shorter working distance (smaller FoV) for imaging the posterior arch. Furthermore, a warm and moist intraoral environment often leads to rapid condensation that complicates imaging. To counteract this issue, an anti-fogging coating on the lens or a heating mechanism to keep the imager warm might be necessary. Finally, the camera needs to move around the teeth in order to capture images from multiple angles. In the case of molar teeth, moving from the anterior to the posterior of the teeth will flip the imaged field vertically. Such a sudden change can disorient the operator and interrupt the imaging process. This issue could be alleviated by incorporating an inertial measurement unit (IMU) within the camera system to estimate the camera orientation and adjust the image accordingly.

The reconstruction algorithm of CPDI relies on subject contrast to triangulate and recover shape. Healthy teeth unfortunately typically lack pronounced visual contrast, which could partly explain the reconstruction errors shown in our studies, particularly on the smooth teeth surface. Surface contrast is determined by the local absorption and scattering properties, which are wavelength dependent. It would be valuable to perform a multispectral analysis of the intrinsic sources of contrast [34], and identify the most effective wavelengths in red, green, and blue spectral bands to maximize the contrast. Another inherent limitation of photogrammetry lies in its inability to recover the absolute physical scale. If a dimensional measurement is required with CPDI, a calibrator with known dimensions can be placed within the scene to serve as a reference [24].

The low-cost nature and ease of use of CPDI will potentially benefit patient care both within dental clinics and home settings. 3D full-color dental models have the potential to replace conventional dental images and augment the text-based descriptions in patient charts. CPDI could also reduce the frequency of dental visits and overall costs by enabling at-home applications [35]. For instance, dental visits after orthodontic procedures could be replaced by providing the 3D dental model generated at home to the dental care providers for progress evaluation. Similarly, a 3D dental model could aid dental examination during remote dental visits.

## 5. Conclusions

In this study, we demonstrated that CPDI is a viable, cost-effective technique for achieving full-color 3D dental imaging. CPDI can generate 3D scans of dental models and in vivo teeth with good representations of their morphology and dental features. Future studies will focus on a comprehensive evaluation of its clinical benefits through a large patient cohort.

## Data Availability

The data that support the findings of this study are available from the corresponding author upon reasonable request.
